# EEG Abnormalities and Phenotypic Correlates in Preschoolers with Autism Spectrum Disorder: A Single-Center Study

**DOI:** 10.3390/jcm14020529

**Published:** 2025-01-15

**Authors:** Luca Ferrini, Emanuele Bartolini, Alice Mancini, Raffaella Tancredi, Anna Rita Ferrari, Sara Calderoni

**Affiliations:** 1 Department of Developmental Neuroscience, IRCCS Stella Maris Foundation, 56128 Pisa, Italy; l.ferrini5@studenti.unipi.it (L.F.); alice.mancini@fsm.unipi.it (A.M.); raffaella.tancredi@fsm.unipi.it (R.T.); annarita.ferrari@fsm.unipi.it (A.R.F.); sara.calderoni@fsm.unipi.it (S.C.); 2 Department of Translational Research and of New Surgical and Medical Technologies, University of Pisa, 56126 Pisa, Italy; 3 Tuscany PhD Programme in Neurosciences, NEUROFARBA Deparment, University of Florence, Viale Pieraccini, 6, 50139 Firenze, Italy; 4 Department of Clinical and Experimental Medicine, University of Pisa, 56126 Pisa, Italy

**Keywords:** autism spectrum disorder (ASD), electroencephalogram (EEG), subclinical electroencephalographic abnormalities (SEAs), subclinical epileptiform discharges (SEDs), neurodevelopmental disorders

## Abstract

**Background:** The literature suggests the existence of an association between autism spectrum disorders (ASDs) and subclinical electroencephalographic abnormalities (SEAs), which show a heterogeneous prevalence rate (12.5–60.7%) within the pediatric ASD population. The aim of this study was to investigate the EEG findings in a cohort of ASD preschoolers and their correlation with the phenotypic characteristics. **Methods:** We retrospectively reviewed data on 141 ASD preschoolers evaluated in a tertiary care university hospital over the period 2008–2018. All participants underwent at least one standard polygraphic electroencephalogram (EEG) and a clinical multidisciplinary assessment with standardized instruments. **Results:** 77 patients (55%) showed SEAs, which were mainly represented by epileptiform discharges (*p* < 0.00001), especially focal and multifocal (*p* = 0.010). Abnormal EEG (*p* = 0.035) and epileptiform discharges (*p* = 0.014) were associated with seizure onset and were predominant in sleep (*p* < 0.00001). Patients with abnormal tracing (*p* = 0.031) and slow abnormalities (*p* < 0.001) were significantly younger. ASD severity was not found to be correlated with EEG results, which showed a potential, albeit non-significant, association with some psychometric parameters. Very similar results were found when patients were divided according to sex. **Conclusions:** EEG abnormalities appear to correlate more with ASD internalizing, externalizing and emotional comorbidities, rather than with ASD core symptoms; larger samples are needed to further investigate this association.

## 1. Introduction

Autism spectrum disorders (ASDs) are a heterogeneous group of neurodevelopmental conditions characterized by persistent deficits in social communication and social interaction across multiple contexts and by the presence of restricted, repetitive patterns of behavior, interests, or activities. The mere presence of the aforementioned clinical elements, however, is not sufficient to make the diagnosis, as the American Psychiatric Association (APA) has established that it is also necessary for them to arise during the early period of development and to cause clinically significant impairment in social, occupational, or other important areas of adaptive functioning (Diagnostic and Statistical Manual of Mental Disorders, 5th Edition, Text Revision [1]). The prevalence of ASD worldwide is estimated to be between 1.09 and 436 cases per 10,000 inhabitants, with a median of 100 cases per 10,000 inhabitants; these are very heterogeneous values, which are affected by various factors non-uniformly distributed throughout the world (i.e., ethnoracial and socioeconomical background, sociodemographic status, and community awareness about autism), responsible for a higher prevalence in Western countries compared, for example, to South-East Asia [1,2,3].

The diagnosis of ASD is made more frequently in males, with a male-to-female ratio varying between 3:1 and 4:1 [1]; this difference is probably due to several aspects, including the use of androcentric diagnostic criteria and tools [4], the phenomenon of camouflaging [5], and the hypothetical protective effect carried out by particular genetic, epigenetic, and hormonal factors [6,7,8].

Autism spectrum disorder, indeed, often occurs in comorbidity with other pathological conditions, among which intellectual disability (ID) is particularly important—it can be detected in 37.9% of autistic children [9] and this inevitably complicates the diagnosis of ASD, for which it is necessary that autistic symptoms must not be explained by a clinical picture of ID [1]. Given their notable frequency in the ASD population, particular attention must be paid to emotional and behavioral disorders too, which can be divided into internalizing disorders, mainly represented by anxiety (30–80%) [10] and depression (11–23%) [11], and externalizing disorders, especially aggressivity (17–56%) [12], hyperactivity, and inattention (13.8–66.7%) [13]. The correct diagnosis and management of these comorbidities is extremely important, as they have a heavy impact on the quality of life of autistic subjects [14]. Other recurrent conditions in the autistic population are represented by specific learning disorder (23.5–67%) [15,16,17], developmental coordination disorder (60–90%) [18,19,20,21], epilepsy (3.9–46%) [22,23,24,25,26,27,28,29,30,31,32,33,34,35,36,37,38,39,40,41,42,43,44,45,46,47,48], sleep disorders (50–80%) [49], and gastrointestinal symptoms (9–91%) [50].

Despite recent advances in genetics and the environmental factors contributing to ASD [8,51], its pathogenesis is still largely unknown today, with the idiopathic form of the disorder still accounting for approximately 85% of the diagnoses [52]. What we know is still broadly the result of various hypotheses. Among the most accredited ones, we have those relating to various possible abnormalities in the development of neuronal circuits [53], which, furthermore, are potentially not mutually exclusive with each other. For example, several authors have observed the presence of an imbalance between neurotransmitters, both excitatory (glutamate) and inhibitory (gamma-aminobutyric acid) [54,55,56,57,58,59], while others have found the existence of anomalous connections between different areas of the brain, in terms of both hypoconnectivity [60], especially between long-range brain regions [61], and hyperconnectivity [62], especially in local circuits [63], giving rise to the hypothesis of ASD as a connectopathy [64]. Other hypotheses concern the abnormal expression of a wide variety of neuronal proteins involved both in neurotransmission, such as the delta subunit of GABA-A receptors (encoded by the *GABRD* gene) [65], Parvalbumin [66,67], and in early brain development; for example, the *CYFIP1* gene (cytoplasmic FMRP-interacting protein 1), the *CHD5* gene (chromodomain helicase DNA-binding protein 5), the *CNTNAP2* gene (contactin-associated protein 2), and other genes coding for neurexins [68,69,70,71]. Lastly, a potential important role for neuroinflammation has recently been suggested [72], with growing interest in the involvement of inflammatory markers such as high-mobility group box-1 protein (HMGB1) and caspase-7 (CASP7), both of which are implicated in neuronal death and found to be higher in autistic subjects than in neurotypical peers [73,74].

In this framework, the literature suggests that ASD shares these neurobiological bases with epilepsy [54,75,76,77,78,79,80,81,82], whose frequency varies depending on several factors heterogeneously distributed within various study cohorts, including the type of electroencephalogram (EEG) study, ASD diagnostic criteria, the co-occurrence of ID, developmental regression, age, gender, and etiology (idiopathic versus non-idiopathic) [83,84,85,86,87]. Since Hans Berger introduced it in 1924, EEG has been extensively used to investigate the autism–epilepsy association—this method records the brain’s electrical activity by measuring it as the total sum of excitatory and inhibitory neuronal postsynaptic potentials [88].

Through the use of EEG, various authors have highlighted the existence of a high recurrence of subclinical electroencephalographic abnormalities (SEAs), such as subclinical epileptiform discharges (SEDs) or paroxysmal slowing activity, not only in children with epilepsy and ASD, but also in those who have not experienced seizures. In the latter, the SEAs show a heterogeneous frequency, ranging from 12.5% to 60.7%, while this figure rises to 66.3% when patients with seizures are included too [83,89,90,91,92,93,94,95]. The role of SEAs in predicting the development of epilepsy in children with ASD is unclear. Despite the current lack of sufficient evidence, numerous authors agree, indeed, that SEAs could also impact neurodevelopmental outcomes [83,95,96,97].

Within this context, the aim of the present retrospective observational study is to depict the electroencephalographic findings observed in children of preschool age with ASD, and to correlate them with the patients’ phenotypic characteristics. Indeed, based on the most recent scientific literature on this topic [98], it is reasonable to assume that the EEG features might show an affinity with the phenotypic profile of the autistic subject. Hence, we expected to identify a relationship between EEG abnormalities and specific clinical traits within our cohort.

## 2. Materials and Methods

### 2.1. Participants

We retrospectively reviewed data on preschool children evaluated between January 2008 and June 2018 in both inpatient and outpatient settings at the IRCCS Stella Maris Foundation (Pisa, Italy), a tertiary care university hospital. Despite this being a single-center study, the catchment area of our facility extends to a large part of Italy and the subjects come from very heterogeneous cultural and socioeconomic backgrounds.

The study population was composed of 141 children, 115 males and 26 females (mean age: 43.91 months, SD: 13.79 months; range: 18–73.32 months). Specifically, in the male population the mean age was 44.74 months (SD: 13.72 months; range: 20.77–70.27 months), while in the female population it was 40.27 months (SD: 13.74 months; range: 18–73.32 months). The mean age reported refers to the age of the participants at the time of their first assessment carried out at our hospital; for some, these data coincide with their age at the diagnosis of ASD, while for others it does not, as this had already been carried out at other centers.

ASD diagnosis was performed or confirmed by a multidisciplinary team (a senior child psychiatrist, an experienced clinical child psychologist, an educational therapist, and a speech and language pathologist) during 5–7 days of extensive evaluation. The subject’s neurodevelopmental profile was investigated in a comprehensive manner, without being limited exclusively to the autistic spectrum. Diagnostic criteria were applied according to the time of the evaluation. Therefore, approximately 1/3 of the patients received a diagnosis of autistic disorder, Asperger’s disorder, or pervasive developmental disorder—not otherwise specified according to the Diagnostic and Statistical Manual of Mental Disorders, 4th Edition, Text Revision (DSM-IV-TR) criteria [99], while the remaining patients evaluated after 2013 received a diagnosis of autism spectrum disorder according to the Diagnostic and Statistical Manual of Mental Disorders, 5th Edition (DSM-5) criteria [1]. For the same reason, the diagnosis was confirmed using the Autism Diagnostic Observation Schedule-Generic (ADOS-G) [100], in approximately 1/3 of the children, or the Autism Diagnostic Observation Schedule-2 (ADOS-2) [101], in the remaining 2/3, using the specific module depending on the patient (Toddler, 1, or 2); due to poor collaboration, in 5 cases (all males) it was not possible to confirm the diagnosis with either of the two assessments.

All children included in this study were 6 years old or younger when they first entered our center and performed at least one standard polygraphic EEG recording in the context of the diagnostic evaluation for ASD. No patient was on psychopharmacological treatment and none of them had already received a diagnosis of epilepsy at the moment of their first EEG. The presence of other neurodevelopmental disorders was not evaluated with standardized tests, due to the age range of our sample, in which it is difficult to distinguish between delay/impairment and disorder (e.g., for developmental language disorder and attention deficit hyperactivity disorder) at least in the youngest children of our sample [102,103]. Any subsequent diagnosis of intellectual disability was taken into account for the interpretation of the results.

The exclusion criteria were: ASD associated with a defined neurological and/or metabolic or genetic condition with a defined etiology (non-idiopathic ASD); EEG recording not evaluable due to severe technical artifacts for poor collaboration.

The absence of a sleep recording was considered when interpreting the results, but did not constitute a reason for exclusion.

The parents of all subjects involved in the present study provided informed consent to use the data collected through clinical investigations and polygraphic EEG recordings for research purposes in an anonymous and aggregated form. All of the procedures complied with the Helsinki Declaration.

### 2.2. Clinical Investigations in ASD Subjects

The participants underwent further clinical assessment in order to complete the definition of the phenotypic profile.

We used the ADOS Calibrated Severity Score (ADOS-CSS) as a clinical measure of ASD severity. According to Lord et al., 2012 [101], by comparing the total score (range: 1–10) with pre-established cut-off values, it is possible to distinguish 4 categories of increasing severity: minimal-to-no evidence (range: 1–2), low levels (range: 3–4), moderate levels (range: 5–7), and high levels (range 8–10) of autistic features. In the present paper, we often refer to ADOS-CSS scores between 5 and 10 as moderate–severe ASD.

To assess the children’s intellectual abilities, several standardized tests were used, tailored to their varying levels of verbal and functional skills. These tests included the Leiter International Performance Scale—Revised (LIPS-R), the Griffiths Mental Development Scales—Extended Revised (GMDS-ER), the Griffiths Scales of Child Development 3rd Edition (Griffiths III) [104], and the Italian version of the Wechsler Preschool and Primary Scale of Intelligence (WPPSI) [105]. For tests that provided a mental age (MA), the IQ was calculated by dividing the mental age by the child’s chronological age (CA) and then multiplying by 100 (MA/CA × 100). In this study, we focused specifically on non-verbal IQ scores, also known as performance IQ (PIQ).

In order to evaluate behavioral and emotional problems, we used the Italian version of the Child Behavior Checklist (CBCL 1½-5), which consists of 100 items regarding the child’s behavior [106,107]. We focused our attention on the Internalizing (a combination of the Emotionally Reactive, Anxious/Depressed, Somatic Complaints, and Withdrawn scales), Externalizing (a combination of the Aggressive Behavior and Attention Problems scales), and Total Problems (which considers all 100 items) summary scales. To interpret the results we referred to both the CBCL summary scales and the CBCL DSM-oriented scales scoring systems, which distinguish three categories of increasing severity: clinical insignificance (t-score ≤ 60 for the CBCL summary scales; t-score ≤ 65 for the CBCL DSM-oriented scales), borderline clinical range (t-score between 60 and 63 for the CBCL summary scales; t-score between 65 and 69 for the CBCL DSM-oriented scales), and clinical significance (t-score ≥ 64 for the CBCL summary scales; t-score ≥ 70 for the CBCL DSM-oriented scales) [106]. Together with these three summary scales, we also took into account the CBCL—Emotional Dysregulation Profile (CBCL-ED), which consists of a set of the Anxiety/Depression scale, Aggression scale, and Attention Syndrome scale. In this case, by comparing the sum of the t-scores of these three scales with other cut-offs, it was possible to distinguish three categories of increasing severity: patients with low/no emotional dysregulation (t-score < 180), patients with deficient emotional self-regulation (t-score between 180 and 210), and patients with severe emotional dysregulation (t-score ≥ 210).

### 2.3. EEG Recording and Analysis

The polygraphic EEG recording was performed after partial sleep deprivation and was aimed to include a period of wakefulness and a period of daytime sleep, as recommended by the guidelines of the International League Against Epilepsy (ILAE) [108]. The EEG recordings were performed non-invasively according to the 10–20 International System at the EPILAB of the IRCCS Stella Maris Foundation (Pisa, Italy), with two surface electromyographic (EMG) electrodes placed over the deltoid muscles of both sides; two experienced neurophysiologists (E.B., A.R.F.) analyzed the recordings in joint sessions. During wakefulness, each patient underwent two activation procedures, represented by hyperventilation (HV) and intermittent photic stimulation (IPS) [109], in order to increase the diagnostic yield. Parents were also recommended to preliminarily perform adequate sleep deprivation [110], both as a further activation procedure and to encourage the child to fall asleep during the second part of the recording.

We focused on the first polygraphic EEG recording for each child. The mean age at the first recording was 43.08 months (SD: 18.71 months; range: 7.04–141.55 months), preceding the formal diagnosis of ASD in a subgroup of patients. In relation to this parameter, females (mean age = 40.25 months; SD: 12.52 months; range: 24.12–72.41 months) were found to be slightly younger than males (mean age = 43.72 months; SD: 19.83 months; range: 7.04–141.55 months). An EEG longitudinal follow-up was available for 46 participants (11 females and 35 males). Each EEG recording was systematically assessed and qualitatively classified according to the definitions provided in the glossary proposed by Kane et al. [111]. Firstly, we categorized the abnormal EEG findings using as classifiers the following terms:

- Focal, multifocal, or diffuse epileptiform discharges (spikes, polyspike discharges, sharp waves, spike-and-slow-wave complexes);
- Non-epileptiform abnormalities, which include:
  ○ Focal or diffuse slow-wave activity (including the slowing of background activity);
  ○ Fast activity.

According to the presence or absence of the aforementioned abnormalities, we, respectively, classified the EEG exam as ‘abnormal’ or ‘normal’. Finally, to define the location of focal and multifocal grapho-elements, we divided the scalp into 3 regions: the frontal midline region, the temporal region, and the posterior region.

### 2.4. Statistical Analysis

We performed the statistical analysis using the R studio software package (version 2024.04.0+735). The binary parameters were tested by the χ^2^ goodness of fit test and the χ^2^ test for categorical variables. The normality of the distribution of quantitative variables within our population was tested using the D’Agostino–Pearson normality test, while the comparison of quantitative variables between groups was performed by an independent samples *t*-test or one-way ANOVA (with Welch’s correction for nonhomogeneous variances), after correlation analysis using Pearson’s correlation coefficient (r). We considered a *p*-value ≤ 0.05 to be statistically significant and we accompanied each statistical result with the corresponding effect size (Cohen’s ω for the χ^2^ goodness of fit test; φ for the χ^2^ test for categorical variables; Cohen’s d for the independent samples *t*-test; ω^2^ for the one-way ANOVA).

## 3. Results

Among the 141 patients recruited for this study, one subject was excluded since his EEG recording was not evaluable due to technical artifacts for poor collaboration. We then included 114 males (81.4%) and 26 females (18.6%), with a M:F ratio of approximately 4.38:1, who showed no significant differences in age at the time of their first evaluation carried out at our hospital (male mean age = 44.77 vs. female mean age = 40.27, *p* = 0.141, Cohen’s d = 0.327). Nine participants (6.4%) experienced seizures (seven males and two females); two subjects (1.4%) had already shown them before the first EEG assessment, while the remaining seven (5%) developed them at a later time.

**Figure 1:**
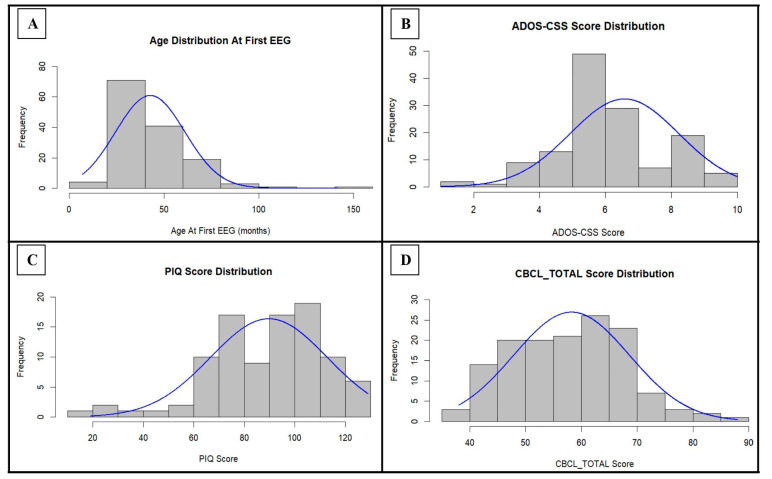
Distribution of age at the first EEG, ADOS-CSS score, PIQ score, and CBCL_TOTAL score within the cohort: for each parameter, the normal curve expected for those values of mean and standard deviation is indicated in blue. ADOS-CSS: ADOS Calibrated Severity Score; PIQ: Performance Intelligence Quotient; CBCL_TOTAL: Child Behavior Checklist—Total Problems Scale.

In order to summarize the composition of the study sample from a clinical point of view, the distribution of the clinical raw scores is reported in Figure 1 and Figure S1 (Supplementary File S1), while the normality testing is shown in Table S1 (Supplementary File S1).

**Figure 2:**
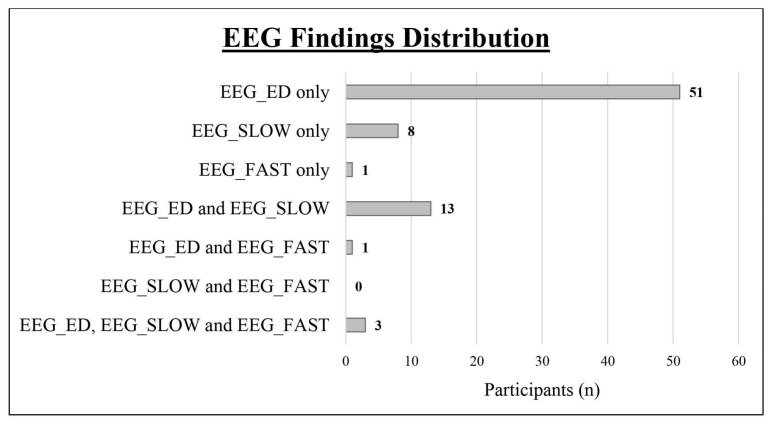
Distribution of the different types of EEG abnormalities within the study cohort. EEG_ED: epileptiform discharges; EEG_SLOW: slow abnormalities; EEG_FAST: fast abnormalities.

We obtained a complete polygraphic EEG recording (wakefulness + sleep) from 128 patients (91.43%), while it was not possible to obtain a sleep recording in the remaining 13 cases (12 males and 1 female). As shown in Table 1 and Figure 2, we found an abnormal EEG in 77 subjects (55%; epileptiform discharges in 68, slow abnormalities in 24, and fast rhythmic activity in 5). Comparing the two sexes, the EEG was abnormal in 17 girls (65.38%; epileptiform discharges in 15, slow abnormalities in 4, fast rhythmic activity in 2) and in 60 boys (52.63%; epileptiform discharges in 53, slow abnormalities in 20, fast rhythmic activity in 3). Epileptiform discharges were always found to be more frequent than slow abnormalities (see Table 1)

We found no significant differences between females and males in terms of the frequency of abnormal EEG (*p* = 0.238, φ = 0.100); this was also true when separately considering epileptiform discharges (*p* = 0.302, φ = 0.087) and slow abnormalities (*p* = 0.792, φ = 0.022). The corresponding contingency tables are shown in Table 2 (see A and B) and Table S2 (Supplementary File S1—see A).

**Figure 3:**
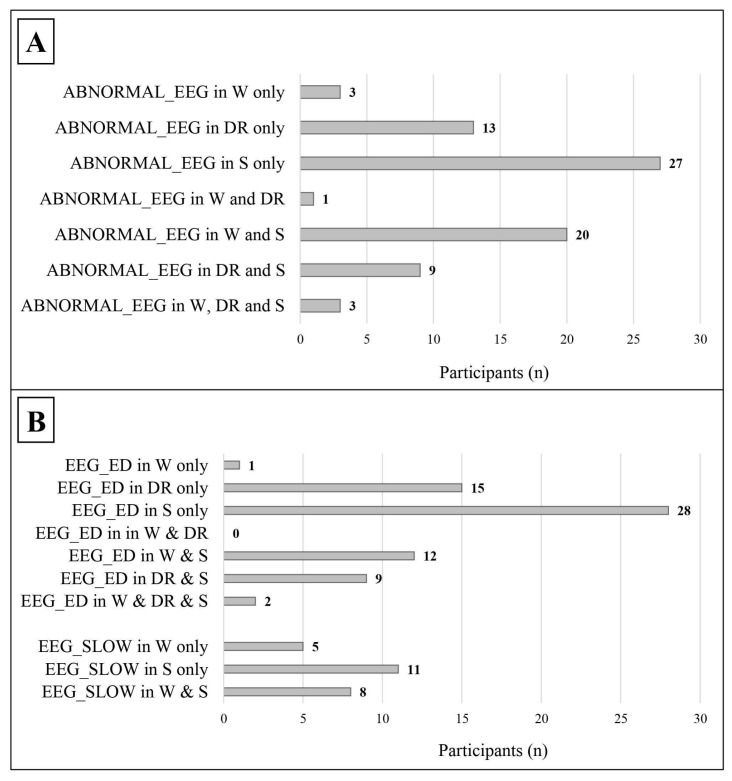
Distribution of abnormal EEG findings in relation to the state of alertness. The data refer only to patients with a complete standard polygraphic EEG (wakefulness + sleep). (**A**) The distribution of abnormal EEG in relation to the state of alertness. (**B**) The distribution of the different types of EEG abnormalities in relation to the state of alertness. ABNORMAL_EEG: abnormal EEG tracing; EEG_ED: epileptiform discharges; EEG_SLOW: slow abnormalities; W: wakefulness; DR: drowsiness; S: sleep.

As shown in Table 3 and Figure 3, leaving out those who showed EEG abnormalities both in wakefulness and in sleep, an abnormal EEG (49 vs. 3) and epileptiform discharges, in particular (52 vs. 1), were more frequent during sleep/drowsiness; these results were confirmed both in males (39 vs. 2 for abnormal EEG; 40 vs. 1 for epileptiform discharges) and females (10 vs. 1 for abnormal EEG; 12 vs. 0 for epileptiform discharges). Slow abnormalities showed a similar tendency in the general group (11 vs. 5, *p* = 0.134, Cohen’s ω= 0.375).

**Figure 4:**
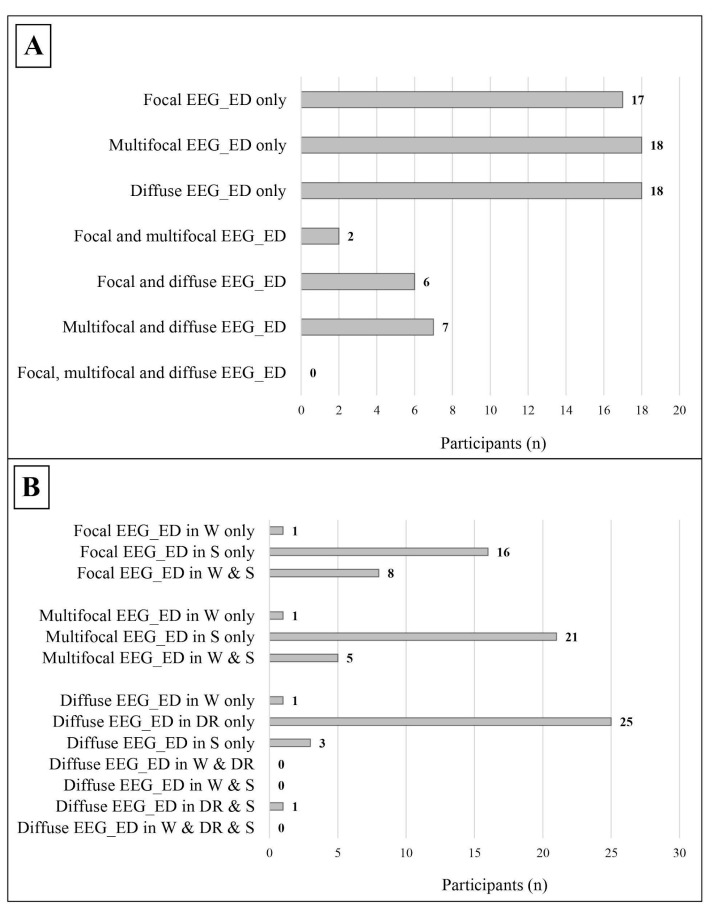
Distribution of the different subtypes of epileptic discharges within the study cohort. (**A**) The distribution of different subtypes of epileptic discharges in relation to each other, the data refer to the whole cohort. (**B**) The distribution of the different subtypes of epileptic discharges in relation to the state of alertness; the data refer only to patients with a complete standard polygraphic EEG (wakefulness + sleep). EEG_ED: epileptiform discharges; W: wakefulness; DR: drowsiness; S: sleep.

We further divided the epileptiform discharges into three subtypes (Table 4 and Figure 4):

- Focal, which were found in 25 participants, mostly during sleep;
- Multifocal, which were found in 27 participants, mostly during sleep;
- Diffuse, which were found in 31 participants, mostly during drowsiness.

Focal/multifocal epileptiform discharges were significantly more frequent than diffuse discharges, both in the general sample (37 vs. 18, *p* = 0.010, Cohen’s ω = 0.345) and in males (29 vs. 13, *p* = 0.013, Cohen’s ω = 0.381). The main sites of epileptiform discharges were midline regions, while slow abnormalities were mostly located in the temporal lobe (see Table 5).

Age at the time of EEG evaluation influenced the outcome of the examination, as subjects with abnormal results were found to be significantly younger (mean age = 39.55 vs. 46.65 months, *p* = 0.031, Cohen’s d = 0.382). These results were statistically significant considering slow abnormalities (mean age = 33.13 vs. 44.73 months, *p* < 0.001, Cohen’s d = 0.761) and showed a similar tendency also for epileptiform discharges (mean age = 40.46 vs. 44.97 months, *p* = 0.144, Cohen’s d = 0.248). These results were confirmed above all in the male subgroup, less so in the female one, in which, by contrast, the age of the patients with an abnormal EEG and epileptiform discharges was higher, although without reaching statistical significance (see Table 6). After excluding the participant with a non-evaluable EEG, no significant difference in terms of age at the first EEG recording was found between the sexes (male mean age = 43.32 vs. female mean age = 40.24, *p* = 0.320, Cohen’s d = 0.188).

Based on the ADOS-CSS score, two participants showed minimal-to-no evidence of autistic features, 10 showed low levels, 91 showed moderate levels, and 31 showed high levels (see Table 7). Considering the whole cohort, an abnormal EEG and epileptiform discharges were less frequent in patients with moderate–severe ASD (ADOS-CSS score ≥ 5), while the opposite was observed when considering slow abnormalities; overall, the EEG findings did not show any association with ASD severity (see Table 8).

With regard to the clinical assessments, as shown in Table S3 (Supplementary File S1), some weak or at most moderate correlations were found between the ADOS-CSS score and the PIQ and CBCL scores, with some differences between the sex subgroups.

Equally, we observed no statistically significant correlation between any of the measured psychometric parameters and the EEG findings (normal/abnormal EEG, any subtype of EEG abnormalities, the location of EEG abnormalities). However, we observed that children with an abnormal EEG tended to have a lower PIQ (mean value = 88.00 vs. 98.00, *p* = 0.059, Cohen’s d = 0.389), as well as slightly worse total CBCL scores (mean value = 59.30 vs. 56.95, *p* = 0.182, Cohen’s d = 0.227) and CBCL-ED scores (mean value = 171.77 vs. 167.78, *p* = 0.154, Cohen’s d = 0.244). Analyzing the type of EEG abnormalities, we found these trends were driven by epileptiform discharges. In fact, patients with epileptiform abnormalities showed a lower PIQ (mean value = 86.44 vs. 92.73, *p* = 0.190, Cohen’s d = 0.272) and slightly worse CBCL-ED scores (mean value = 171.96 vs. 168.04, *p* = 0.159, Cohen’s d = 0.239), while their scores at the externalizing problems CBCL were higher (mean value = 55.68 vs. 53.87, *p* = 0.197, Cohen’s d = 0.219). Conversely, we found no notable association with slow or fast abnormalities. The analysis of the individual female and male subgroups revealed results that were quite in line with those just described, albeit with some differences (see Table 9):

The ADOS-CSS score and psychometric scores were found to be quite homogeneous in the two subgroups (see Table 10), with only the PIQ being significantly lower among females (mean value = 75.33 vs. 94.54, *p* = 0.004, Cohen’s d = 0.790).

Furthermore, we separately analyzed the effects of the EEG findings in wakefulness and sleep on the measured psychometric parameters. No statistically significant correlation was found for the EEG abnormalities during wakefulness. Instead, we disclosed that patients with an abnormal EEG during sleep still tended to have lower scores for the PIQ (mean value = 85.01 vs. 93.46, *p* = 0.100, Cohen’s d = 0.357), but this tendency was not confirmed in the subgroups with epileptiform discharges (mean value = 86.70 vs. 91.31, *p* = 0.388, Cohen’s d = 0.191) and slow abnormalities (mean value = 83.38 vs. 90.57, *p* = 0.333, Cohen’s d = 0.297). By repeating these analyses separately for the two sexes (see Table 11), only in females did we observe a significantly lower PIQ in the event of an abnormal EEG during sleep (mean value = 61.33 vs. 90.45, *p* = 0.013, Cohen’s d = 1.127) and a tendency towards a lower PIQ in the presence of epileptiform discharges during sleep (mean value = 64.36 vs. 85.25, *p* = 0.088, Cohen’s d = 0.747).

Taking into consideration the location of EEG abnormalities (see Table 5), focal/multifocal epileptiform discharges were observed mostly in the frontal midline region (36.76%), while the posterior site was the least frequently affected (4.42%). On the other hand, slow abnormalities were mainly located in the temporal (50%) and posterior (37.50%) regions, with the frontal midline site affected only in three cases (12.50%). Since the subgroup of children with slow abnormalities was too small to be analyzed, we focused on the location of epileptiform abnormalities, which showed no correlation with any of the clinical scores (see Table 12). The results obtained in the general sample were also confirmed among males, while females were not evaluable due to the lack of participants.

Lastly, seizures were significantly associated with the presence of an abnormal EEG (*p* = 0.035, φ = 0.179) and especially of epileptiform discharges (*p* = 0.014, φ = 0.208); no association was found with slow abnormalities (*p* = 0.620, *φ* = 0.042). The corresponding contingency tables are shown in Table 2 (see C and D) and Table S2 (Supplementary File S1—see B). Temporal epileptiform discharges during sleep and midline and diffuse epileptiform discharges during both wakefulness and sleep were found in the two subjects who already had seizures at the time of their first evaluation at our center. Of the remaining 66 participants with subclinical epileptiform discharges, 6 (9.1%) developed seizures during follow-up; by contrast, only 1 (1.4%) of the 72 subjects without subclinical epileptiform discharges developed seizures later on in the study. Albeit with the limit of their low overall frequency, these data show that seizures occurred significantly more prevalently in those who already exhibited SEDs (*p* = 0.039, φ = 0.175).

## 4. Discussion

### 4.1. Relationship Between SEAs and Clinical Features

The main objective of this study is to analyze the relationship between the EEG findings and clinical characteristics of ASD preschoolers.

Focusing on the core symptoms of autism, the ADOS-CSS score is unevenly distributed within our sample, with a poor representation of milder cases (see Figure 1 and Table 7); in fact, the majority (91.04%) are placed in the medium–high severity range (ADOS-CSS score ≥ 5). This aspect can be explained by the fact that our population belongs to the user base of a tertiary care university hospital, where overall, more severe cases tend to be admitted; for this reason, the study population may not be representative of the full spectrum of ASD, thus limiting the generalizability of the results. Despite this potential bias, the composition of our sample, in terms of the severity of the autistic phenotype, is very similar to those of other studies concerning ASD, such as that by Pellicano et al. [112]. Contrary to what has been reported in several studies in the literature on autism [96,113,114,115], within our population an abnormal EEG pattern was found more frequently among participants with ADOS-CSS score ≤ 4, with the exception of the male subgroup (see Table 8). Despite this, no connection between the SEAs and the severity of autistic core symptoms seems to be present. This same conclusion was also reached by Anukirthiga et al. [115] and Milovanovic et al. [89], while other authors found a statistically significant association between EEG abnormalities and the severe autistic phenotype [96,113,114,116]. In particular, Yousef and colleagues [114] reported the presence of an interesting link between the severity of autistic symptoms and the location of EEG abnormalities (severe cases showed mostly bicentrotemporal, bitempofrontal, and frontotemporal abnormalities), which did not find any confirmation within our cohort (see Table 12).

Regarding concomitant psychopathological symptoms, the psychometric parameters we measured show a rather homogeneous distribution within the general sample (see Figure 1). When comparing the two sexes (see Table 10), however, PIQ was significantly lower among females (*p* = 0.004). In agreement with this result, several studies [117,118,119,120,121], especially older ones, have highlighted a lower full-scale IQ in ASD females, while some more recent investigations do not confirm these data [122,123,124]. Potentially, this significant difference in PIQ that we detected could be the result of the tendency to late diagnosis of ASD in females [5,125]. In fact, for some autistic females, having better cognitive abilities can lead to a delay in the diagnosis of ASD [120,126,127], a phenomenon that, in the case of young samples like ours, predisposes them to a recruitment bias in favor of patients with lower IQ scores. In the most recent scientific literature on autism [98], the relationship between ID/full-scale IQ and electrical brain activity has been the subject of great interest, providing conflicting results. Some studies [128] argue that there is no association between abnormal EEG patterns and ID, in agreement with Akhter [129]. The latter investigation, however, also observed a significantly higher frequency of EEG abnormalities in subjects with moderate–severe ID, in agreement with Nicotera et al. [96] and Anukirthiga et al. [115]. In the current study, PIQ was notably lower in case of abnormal EEG (*p* = 0.059), particularly in females (*p* = 0.099), and in SEDs (*p* = 0.190). This relationship would seem to be driven by sleep abnormalities (see Table 11), which would play a significant role both in the general sample and, above all, in the female subgroup, in which PIQ was significantly lower in the presence of abnormal EEG (*p* = 0.013).

As for CBCL scores, in the present study we did not detect any significant difference between sexes (see Table 10), in agreement with Pisula et al. [130] and Muratori et al. [131]. However, the psychopathological comorbidities of ASD are still the subject of heated debate. Contrary to our results, several authors have highlighted the greater prevalence of internalizing disorders in females [132,133,134,135,136] and externalizing disorders in males [134,135,137,138,139], while others have reported conflicting results [140,141,142,143,144]. From the point of view of their relationship with EEG findings, the most recent scientific literature has focused mainly on externalizing disorders, also in this case providing contrasting views, both in favor [91,96] and against [97,145] the presence of an association. Valvo et al. [146] and Capal et al. [95], referring to the CBCL scores, carried out a more complete investigation, which highlighted the absence of an association with the EEG for both internalizing and externalizing symptoms. Similarly, we also did not find any statistically significant associations in the present study, but we identified potential trends that appear to link SEAs to the scores of all four CBCL scales we considered (see Table 9). The strongest trend, in particular, concerns externalizing symptoms, which were more severe in females with an abnormal EEG (*p* = 0.052), despite remaining on average below the threshold. A weaker relationship was found for CBCL total and CBCL-ED scores (see Table 9), both higher in participants with EEG abnormalities, especially in females. Also, in this case, the mean scores remained sub-threshold, with the exception of the mean CBCL total score in females with SEAs (mean value = 61.00), which is considered clinically borderline by the CBCL summary scales, though remaining clinically non-significant according to the CBCL DSM-oriented scales. Considering the slow abnormalities and the SEDs separately, the latter were found to be associated, albeit poorly, with worse, but still subclinical, mean scores totalized in the externalizing symptoms and emotional regulation scales (see Table 9). The only trend involving internalizing disorders concerned slow abnormalities, which appeared weakly linked to averagely worse symptoms in males (*p* = 0.175); the mean score remained clinically non-significant, at most borderline according to the CBCL summary scales.

Although statistical significance was not reached in almost all cases, the PIQ and CBCL scores showed an overall fascinating association with EEG findings, which was completely absent, on the contrary, in the case of the ADOS-CSS score (see Table 8 and Table 9). Based on these results, which outline simple, albeit interesting, trends, the presence of SEAs seems to impact more on the cognitive, emotional, and behavioral problems associated with ASD, rather than on its core symptoms. The underlying mechanisms are potentially attributable to the impact that SEAs, especially SEDs, would have on neuroplasticity, but it remains complex to interpret why the effects would be more evident for comorbidities, rather than for autism itself. Further research is needed, as several items of evidence suggest a role for SEAs in the pathogenesis of ASD (see Section 4.3), but in view of these results it cannot be excluded that they may represent more an expression of the alterations of the neuronal circuits, rather than a concause. Given the frequent finding of EEG abnormalities even in non-autistic subjects with ADHD, aggressive behavior, or anxiety symptoms [147,148], at least in a subgroup of patients SEAs could play the role of a pathogenetic bridge between ASD, of which they could represent a consequence, and its comorbidities, towards which they could potentially act as a contributory cause.

### 4.2. Frequency of SEAs

Numerous authors have investigated the interesting relationship between ASD and subclinical EEG abnormalities, with conflicting findings [86,87,93,98]. In our study, SEAs were found in 55% of participants. Such a figure might be an underestimate, as the EEG traces of children with ASD may be burdened by a remarkable number of artifacts that limit the effective duration of the interpretable recording. Even though most of the abnormalities can already be detected during the first 20 min of recording, by simply reaching a duration of 40 min it is possible to increase the yield by 11% [98]. Moreover, obtaining a sleep recording in ASD may be cumbersome; 13 patients (8.57%) in our cohort did not fall asleep during the EEG test. This increases the probability of false negatives, since SEAs tend to occur more frequently during sleep, as formerly observed in young children with typical development [149] and as confirmed in young autistic individuals by our findings and those of other studies [91,93,96,150]. In support of this hypothesis, it is relevant to underline that only one participant of those with a waking-only EEG had an abnormal EEG tracing.

The literature on the relationship between autism and EEG tends to focus mainly on the middle childhood (6–11 years) and young teen (12–14 years) age groups [98], in some cases including adults too [91,95,96,146,151], while only a few papers have focused their attention on preschoolers [152,153]. Former studies have reported very heterogeneous figures of SEAs in autistic children (8–60.7%) [98], compared to which our results are shifted towards the upper end. This is potentially attributable to the reduced age of the participants at the time of the first EEG recording (range: 7.04–141.55 months), since the rate of SEAs tends to progressively decrease with age, especially towards adolescence/adulthood [154,155,156]. Confirming this trend, in our sample it was possible to find a significantly higher frequency of SEAs in the younger participants (see Table 6).

The M:F ratio in our population was 4.38:1, roughly in line with the rest of the scientific literature on autism (4:1) [2,9,157], while the most recent studies that have investigated the association between EEG findings and ASD showed an M:F ratio ranging between 1.3:1 and 5.6:1, with an average of 2.95:1 [98]. Considering only our participants with abnormal EEG, the M:F ratio increases (3.53:1), similarly to what is reported in other investigations [113,128,158]. Regardless of their small number, SEAs were more frequent in the female subgroup rather than in the male one (65.38% vs. 52.63%), in agreement with what has been reported by previous investigations [113,128,158]. However, this difference does not seem to have any statistical significance (*p* = 0.238), as also reported by other studies [113,114,158]. Given the contradictory nature of these results, it is reasonable to further investigate this aspect in order to systematically address a potential gender imbalance and to investigate the underlying pathophysiology [4,5,159].

### 4.3. Relationship Between SEDs and ASD Pathogenesis

Within our sample, subclinical epileptiform discharges were more frequent than slow abnormalities (see Table 1), in agreement with several other authors [83,90,91,160,161,162]. These data are quite interesting, since SEDs seem to have a greater impact on the cognitive processes of the autistic subject than slow abnormalities [97]. Although epileptiform discharges, even when subclinical, are generally considered a non-specific sign of cortical dysfunction, Santarone et al. [152] argue that in autism they may be considered a neurophysiological marker of disease. El Achkar and Spence [163] go further, as they hypothesize that SEDs could contribute to determining the autistic phenotype. According to some authors, in fact, epileptiform discharges would be significantly associated with an anomalous brain development during the first year of life [83,96].

Neuronal hypersynchrony could be involved in one of the most important hypotheses regarding the physiopathology of ASD, which postulates an alteration of the balance between excitatory and inhibitory neurotransmission as the protagonist [164,165]. In studies conducted on humans [166] and on animal models [167], epileptiform discharges, even of short duration, provided that they are recurrent, have been found to be able to induce both neuronal death and axonal sprouting, responsible for the genesis of aberrant circuits characterized by alterations in GABAergic transmission [168,169], and in the levels of cerebral parvalbumin [170,171]. These anomalies are the cause of an excitation/inhibition imbalance, which may contribute to the neurobiology of autism [66,67], of some of its comorbidities (i.e., depression and anxiety) and of epileptic seizures [172].

In our sample, seizures were poorly represented, yet significantly associated with SEAs (*p* = 0.035), highlighting a particularly strong link with SEDs (*p* = 0.014), which were found to be predominantly focal and multifocal, both in the whole population (*p* = 0.010) and in the male subgroup (*p* = 0.013). Similar data, although in the absence of statistical significance, have been previously reported in some [95,96,97,158] but not in other [115,129,152] studies. The focal/multifocal distribution of SEDs could be related to a dysfunction of scattered cerebral hubs rather than to an impairment of the whole brain. It is likely that different neurobiological phenomena promote various EEG features in ASD. An asymmetry of brain morphometry has formerly been reported [173,174,175,176], and it would be noteworthy to explore a relationship between this parameter and SEA distribution. From this point of view, in line with another important hypothesis, which conceives autism as a connectopathy [60,61,63,64], Donovan and Basson [177] hypothesized that ASD could be due to aberrations in the neuronal circuits of specific cortical and subcortical structures. In support of this, Postema et al. [178] and Sha et al. [179] highlighted the presence of an interhemispheric asymmetry in the cortical thickness of the frontal and temporal lobes, which could be attribute to underlying anomalies in neuronal connectivity [179]. These same regions were the most frequently involved by SEDs within our sample (see Table 5), variably in agreement with what has been described in many other studies in the literature [48,93,94,95,96,97,113,128,129,151,152,156,158,180,181,182], suggesting their possible role in the altered brain asymmetry observed in autism. Indeed, it is believed that an abnormal functioning of the frontal and temporal lobes is crucially implicated in the physiopathology of ASD [183,184,185,186,187,188,189].

Noteworthy is the fact that we found most EEG abnormalities during sleep (see Figure 3). Within the scientific literature, the idea that sleep is strictly connected to neuroplasticity is generally shared [190]; during sleep, in fact, it is believed that synapses are subjected to pruning and refinement processes, aimed at optimizing neuronal circuits [191]. A particular role seems to be played by the slow-wave activity (SWA) of non-rapid eye movement (NREM) sleep, as proposed in the synaptic homeostasis hypothesis [192,193,194]. Similarly, sleep spindles also seem to be involved in synapse consolidation [195,196]. Although memory represents, to date, the main function modulated by sleep [197], some authors believe that other cognitive functions may also be influenced by the typical brain activity of NREM sleep phases [198,199,200]. Given these assumptions, it is reasonable to hypothesize that the presence of frequent subclinical epileptiform discharges during sleep may interfere with brain connectivity. This effect would be expressed through a dual action of SEDs, both direct, by interfering themselves with synaptic plasticity [201], and indirect, by perturbing the SWA in the performance of its functions. Also, considering the notable frequency of sleep disorders in the autistic population [202], what has just been described could represent a further point of contact between SEDs and the pathogenesis of ASD, whose ambiguous relationship with sleep is still a matter of debate [98]. These considerations have clinical implications, as they suggest the usefulness of implementing the recording of an EEG tracing during sleep in the diagnostic work-up of autism spectrum disorders. As also shown by other studies [91,93,96,150], sleep tracings make it possible to increase the probability of identifying EEG abnormalities, especially through prolonged recordings [83,91], which could also be performed overnight in case of suspected concomitant sleep disorders.

In light of the potential role of epileptiform discharges in the physiopathology of ASD, several authors have analyzed the possibility of using anti-seizure medications (ASMs) to treat SEDs. During ASM therapy, in fact, SEDs tend to wane [93,203] and some have reported an improvement of the core symptoms [204,205] and behavioral comorbidities of autism [204] too. However, a disease-modifying effect of ASMs has never been demonstrated either in people with epilepsy or in those with ASD, and a pharmaceutical treatment should only be established after epilepsy sets in or in specific encephalopathy settings. A purely preventive treatment of ASD patients with abnormal EEG in the absence of seizures is not currently recommended and remains a highly controversial issue [86,206], with some authors [152] suggesting that it might be justifiable to consider ASMs in case the discharges occupy a significant proportion of NREM sleep in the presence of an arrest of neurocognitive development. Nevertheless, subclinical EEG abnormalities could be related to a different disease course: it has been proposed that the presence of subclinical EEG abnormalities in autistic preschoolers suggests a worse clinical development [97]. Prospective studies would be needed to carefully assess the role of EEG findings as biomarkers of prognosis. Considering the higher frequency of SEAs in younger subjects, it is desirable to continue research in this direction, since the demonstration of the existence of a possible pathogenic relationship between SEDs and autism could allow clinicians to stratify the patients and tailor their treatment more accurately.

### 4.4. Study Limitations

The population size (n = 140) was limited by the difficulty of finding ASD patients with at least one EEG tracing in a natural observational setting. In the general population, EEG in ASD is performed only after seizure onset, which occurs mostly during adolescence [30,207]. However, we often obtain an early EEG exam in newly diagnosed ASD, in order to have a more comprehensive assessment of the patient’s profile. Sharing our observations with other third-level centers could help increase the study population, with a net benefit on the power of statistical analysis [208]. At least a part of the trends that we found by analyzing the relationship between psychometric parameters and EEG could actually hide an actual statistical significance, which could potentially be revealed by repeating the analysis on a larger population. Larger samples are required to detect smaller effects [209] and to counteract the overestimation of the population effect size, which is a common occurrence in small samples [210]. On the other hand, using small cohorts makes it possible to limit bias and measurement errors, improving the overall quality of the data, which can be analyzed with greater care, thus potentially producing more truthful results [209]. In addition, a meta-analysis performed on several small studies that point in the same direction, rather than a single study conducted on a large sample, could lead to a more robust conclusion [209].

However, a further weakness of small samples, which has a significant weight in autism studies, is their poor ability to mitigate the effect of confounding factors [209]. When we analyzed our participants with epileptiform discharges, we were forced, for reasons of statistical power, to group together both those who actually only showed SEDs and those who showed both SEDs and slow abnormalities (see Figure 2). This issue predisposes the study to the risk of having analyzed a spurious correlation, since, in this context, slow abnormalities could have acted as a confounding factor. A similar argument can be made for patients with slow abnormalities (see Figure 2), in which, vice versa, epileptiform discharges could have acted as a confounding factor. For the same reasons, it was not possible to separately analyze the patients with EEG abnormalities exclusively in wakefulness, on the one hand, and those with EEG abnormalities exclusively in sleep, on the other (see Figure 3). To limit the effect of confounding factors, especially when numerous and difficult to control, it is preferable to use a large sample [209]; from this perspective, autism represents an emblematic case, being a complex disorder closely intertwined both pathogenetically and epidemiologically with numerous other neuropsychiatric conditions, often still subclinical or underdiagnosed at preschool age, but already able to influence the results. Although the aim of this study is not to demonstrate causal relationships, for interpretative purposes it is important to point out the weaknesses of the correlations we analyzed, especially in the female subgroup.

Two additional vulnerable points are represented by the tertiary care university hospital selection bias, which has already been exhaustively discussed (see Section 4.1), and the absence of a control group of typically developing children, which precludes the possibility of excluding that what was observed in autistic subjects might also be confirmed in the general population.

Despite these limitations, we strongly believe that the results presented in this manuscript provide important information on the relationship between ASD and electrical brain activity, which certainly need to be further explored using larger, deeply phenotyped samples.

## 5. Conclusions

The analysis of EEG features might represent a potentially important resource in the field of autism spectrum disorders.

Hopefully, the interesting relationship between autism and SEAs could constitute a valid future ally for ASD diagnosis, at least in a subgroup of patients. Our results did not highlight any association between EEG abnormalities and the core symptoms of autism. However, given their notable frequency in the autistic population [83,89,90,91,92,93,94,95], there might be a common underlying pathophysiological background. Our results showed that subclinical EEG abnormalities are frequent in younger children with ASD, suggesting that the age group between 3 and 5 years might be the most suitable for investigation of potential electroencephalographic biomarkers of ASD. Larger studies in this age range, with higher statistical power, could unveil hidden pure associations between the autistic phenotype and the type of EEG abnormalities. Moreover, more space within the samples should be dedicated to autistic females, typically penalized in terms of representation in the literature on autism.

The study of the relationship between SEAs and ASD, however, could also hide possible prognostic advantages, as our data show that EEG abnormalities, and SEDs in particular, seem to be associated with seizures and the development of cognitive and behavioral comorbidities. Given also the potential impact of sleep-related SEAs on neuroplasticity, an early analysis of the EEG tracing, especially during sleep, could make it possible to better frame the autistic patient in the future, potentially assisting the clinician in setting up a therapeutic plan to manage early possible complications of ASD.

## Figures and Tables

**Table 1:** Main electroencephalographic findings. n: number of participants.

	Total (n)	Females (n)	Males (n)
Abnormal EEG	77	17	60
Epileptiform Discharges	68	15	53
Slow Abnormalities	24	4	20
Fast Abnormalities	5	2	3

**Table 2:** Contingency tables for χ^2^ tests regarding the relationship between EEG findings and both sex on the left (see **A** and **B**) and seizures on the right (see **C** and **D**). TOT: total; F: female; M: male; EEG_ED: epileptiform discharges.

**A**				**C**			
	**Normal** **EEG**	**Abnormal** **EEG**	**TOT**		**Normal** **EEG**	**Abnormal** **EEG**	**TOT**
F	9	17	26	No seizures	62	69	131
M	54	60	114	Seizures	1	8	9
TOT	63	77	140	TOT	63	77	140
**B**				**D**			
	**Absence of** **EEG_ED**	**Presence of** **EEG_ED**	**TOT**		**Absence of** **EEG_ED**	**Presence of** **EEG_ED**	**TOT**
F	11	15	26	No seizures	70	61	131
M	61	53	114	Seizures	1	8	9
TOT	72	68	140	TOT	71	69	140

**Table 3:** Main electroencephalographic findings in relation to the state of alertness. n: number of participants; TOT: total; M: males; F: females; EEG_ED: epileptiform discharges; EEG_SLOW: slow abnormalities.

	Wakefulness (n)	Drowsiness (n)	Sleep (n)
Abnormal EEG—TOT	28	26	59
Abnormal EEG—M	21	19	47
Abnormal EEG—F	7	7	12
EEG_ED—TOT	16	26	51
EEG_ED—M	13	19	40
EEG_ED—F	3	7	11
EEG_SLOW—TOT	13	/	19
EEG_SLOW—M	9	/	17
EEG_SLOW—F	4	/	2

**Table 4:** Main epileptiform discharge subtypes: the percentages refer to the total number of participants reporting epileptiform discharges. EEG_ED: epileptiform discharges; n: number of participants; TOT: total; M: males; F: females.

	Complete EEG (n)	Wakefulness (n)	Drowsiness (n)	Sleep (n)
Focal—TOT	25 (36.76%)	9	/	24
Focal—M	21	7	/	20
Focal—F	4	2	/	4
Multifocal—TOT	27 (39.71%)	6	/	26
Multifocal—M	21	5	/	20
Multifocal—F	6	1	/	6
Diffuse—TOT	31 (45.59%)	2	26	4
Diffuse—M	24	2	19	3
Diffuse—F	7	0	7	1

**Table 5:** Distribution by the location of the main EEG abnormalities: the percentages refer to the total number of participants reporting epileptiform discharges or slow abnormalities, respectively. The *+* indicates the occurrence in the same participant of both diffuse and focal/multifocal (in the midline or temporal region) epileptiform discharges. n: number of participants; F/M: focal/multifocal; EEG_ED: epileptiform discharges; EEG_SLOW: slow abnormalities.

	Total (n)	Females (n)	Males (n)
Participants with EEG_ED	68 (100%)	15	53
F/M EEG_ ED in frontal-midline region	25 (36.76%)	6	19
F/M EEG_ ED in temporal region	9 (13.24%)	1	8
F/M EEG_ ED in posterior region	3 (4.42%)	1	2
Diffuse EEG_ ED	20 (29.41%)	5	15
F/M EEG_ ED in frontal-midline region + Diffuse EEG_ ED	6 (8.82%)	0	6
F/M EEG_ ED in temporal region + Diffuse EEG_ ED	5 (7.35%)	2	3
Participants with EEG_SLOW	24 (100%)	4	20
F/M EEG_SLOW in frontal-midline region	3 (12.50%)	0	3
F/M EEG_SLOW in temporal region	12 (50.00%)	0	12
F/M EEG_SLOW in posterior region	9 (37.50%)	4	5

**Table 6:** Correlation between age at first EEG recording and EEG findings (* indicates statistically significant results; **^#^** indicates potential trends): 0 = indicates the absence of the corresponding abnormal EEG finding; 1 = indicates the presence of the corresponding abnormal EEG finding. p: p-value; ABNORMAL_EEG: abnormal EEG tracing; AGE_EEG: age at the first EEG recording; EEG_ED: epileptiform discharges; EEG_SLOW: slow abnormalities.

	General		Females		Males	
p(ABNORMNAL_EEG, AGE_EEG)	0.031 *		0.699		0.018 *	
Cohen’s d(ANORMAL_EEG, AGE_EEG)	0.382		0.168		0.457	
Mean Age at first EEG recording	0 = 46.65	1 = 39.55	0 = 38.79	1 = 41.02	0 = 47.96	1 = 39.14
p(EEG_ED, AGE_EEG)	0.144 ^#^		0.305		0.095 ^#^	
Cohen’s d(EEG_ED, AGE_EEG)	0.248		0.424		0.310	
Mean Age at first EEG recording	0 = 44.97	1 = 40.46	0 = 37.15	1 = 42.52	0 = 46.05	1 = 40.17
p(EEG_SLOW, AGE_EEG)	<0.001 *		0.017 *		0.001 *	
Cohen’s d(EEG_SLOW, AGE_EEG)	0.761		1.406		0.697	
Mean Age at first EEG recording	0 = 44.73	1 = 33.13	0 = 42.32	1 = 28.84	0 = 45.30	1 = 33.99

**Table 7:** Study cohort classification based on ASD severity assessed with ADOS-CSS. ASD: autism spectrum disorder; ADOS-CSS: ADOS Calibrated Severity Score; n: number of participants.

	General (n)	Females (n)	Males (n)
Minimal-to-no evidence of autistic features (ADOS-CSS score range: 1–2)	2	1	1
Low levels of autistic features (ADOS-CSS score range: 3–4)	10	3	7
Moderate levels of autistic features (ADOS-CSS score range: 5–7)	91	18	73
High levels of autistic features (ADOS-CSS score range: 8–10)	31	4	27

**Table 8:** Relationship between ADOS-CSS and abnormal EEG findings: 0 = indicates the absence of the corresponding abnormal EEG finding; 1 = indicates the presence of the corresponding abnormal EEG finding. f: frequency; ADOS-CSS: ADOS Calibrated Severity Score; ABNORMAL_EEG: abnormal EEG tracing; EEG_ED: epileptiform discharges; EEG_SLOW: slow abnormalities.

	General		Females		Males	
f(ABNORMAL_EEG when ADOS-CSS score ≤ 4)	58.33%		75.00%		50.00%	
f(ABNORMAL_EEG when ADOS-CSS score ≥ 5)	54.92%		63.63%		53.00%	
f(EEG_ED when ADOS-CSS score ≤ 4)	58.33%		75.00%		50.00%	
f(EEG_ED when ADOS-CSS score ≥ 5)	47.54%		54.54%		46.00%	
f(EEG_SLOW when ADOS-CSS score ≤ 4)	8.33%		0%		12.50%	
f(EEG_SLOW when ADOS-CSS score ≥ 5)	18.85%		18.18%		19.00%	
p(ABNORMAL_EEG, ADOS-CSS)	0.714		0.737		0.914	
Cohen’s d(ABNORMAL_EEG, ADOS-CSS)	0.064		0.149		0.021	
Mean ADOS-CSS score as regards ABNORMAL_EEG	0 = 6.63	1 = 6.53	0 = 6.56	1 = 6.23	0 = 6.65	1 = 6.61
p(EEG_ED, ADOS-CSS)	0.994		0.824		0.953	
Cohen’s d(EEG_ED, ADOS-CSS)	0.001		0.091		0.011	
Mean ADOS-CSS score as regards EEG_ED	0 = 6.573	1 = 6.576	0 = 6.45	1 = 6.27	0 = 6.64	1 = 6.62
p(EEG_SLOW, ADOS-CSS)	0.250		0.870		0.231	
Cohen’s d(EEG_SLOW, ADOS-CSS)	0.231		0.069		0.278	
Mean ADOS-CSS score as regards EEG_SLOW	0 = 6.64	1 = 6.29	0 = 6.36	1 = 6.25	0 = 6.70	1 = 6.30

**Table 9:** Correlation between psychometric parameters and EEG findings (**^#^** indicates potential trends): 0 *=* indicates the absence of the corresponding abnormal EEG finding; 1 = indicates the presence of the corresponding abnormal EEG finding. ABNORMAL_EEG: abnormal EEG tracing; EEG_ED: epileptiform discharges; EEG_SLOW: slow abnormalities; PIQ: Performance Intelligence Quotient; CBCL_INT: Child Behavior Checklist—Internalizing Problems Scale; CBCL_EXT: Child Behavior Checklist—Externalizing Problems Scale; CBCL_TOTAL: Child Behavior Checklist—Total Problems Scale; CBCL_ED: Child Behavior Checklist—Emotional Dysregulation Profile.

	General		Females		Males	
p(ABNORMAL_EEG, PIQ)	0.059 ^#^		0.099 ^#^		0.532	
Cohen’s d(ABNORMAL_EEG, PIQ)	0.389		0.719		0.149	
Mean PIQ score as regards ABNORMAL_EEG	0 = 98.00	1 = 88.00	0 = 88.00	1 = 69.00	0 = 95.97	1 = 93.15
p(EEG_ED, PIQ)	0.190 ^#^		0.453		0.662	
Cohen’s d(EEG_ED, PIQ)	0.272		0.314		0.106	
Mean PIQ score as regards EEG_ED	0 = 92.73	1 = 86.44	0 = 80.60	1 = 71.57	0 = 95.43	1 = 93.40
p(EEG_SLOW, CBCL_INT)	0.307		0.971		0.175 ^#^	
Cohen’s d(EEG_SLOW, CBCL_INT)	0.245		0.025		0.339	
Mean CBCL_INT score as regards EEG_SLOW	0 = 59.92	1 = 62.38	0 = 60.14	1 = 59.75	0 = 59.86	1 = 62.90
p(ABNORMAL_EEG, CBCL_EXT)	0.223		0.052 ^#^		0.586	
Cohen’s d(ABNORMAL_EEG, CBCL_EXT)	0.209		0.847		0.102	
Mean CBCL_EXT score as regards ABNORMAL_EEG	0 = 53.81	1 = 55.52	0 = 50.78	1 = 56.76	0 = 54.31	1 = 55.17
p(EEG_ED, CBCL_EXT)	0.197 ^#^		0.193 ^#^		0.419	
Cohen’s d(EEG_ED, CBCL_EXT)	0.219		0.548		0.153	
Mean CBCL_EXT score as regards EEG_ED	0 = 53.87	1 = 55.68	0 = 52.27	1 = 56.47	0 = 54.16	1 = 55.45
p(ABNORMAL_EEG, CBCL_TOTAL)	0.182 ^#^		0.133 ^#^		0.474	
Cohen’s d(ABNORMAL_EEG, CBCL_TOTAL)	0.227		0.650		0.135	
Mean CBCL_TOTAL score as regards ABNORMAL_EEG	0 = 56.95	1 = 59.30	0 = 54.00	1 = 61.00	0 = 57.44	1 = 58.82
p(ABNORMAL_EEG, CBCL_ED)	0.154 ^#^		0.124 ^#^		0.346	
Cohen’s d(ABNORMAL_EEG, CBCL_ED)	0.244		0.647		0.178	
Mean CBCL_ED score as regards ABNORMAL_EEG	0 = 167.78	1 = 171.77	0 = 162.11	1 = 172.18	0 = 168.72	1 = 171.65
p(EEG_ED, CBCL_ED)	0.159 ^#^		0.478		0.202	
Cohen’s d(EEG_ED, CBCL_ED)	0.239		0.289		0.241	
Mean CBCL_ED score as regards EEG_ED	0 = 168.04	1 = 171.96	0 = 165.91	1 = 170.73	0 = 168.43	1 = 172.38

**Table 10:** Comparison between female and male subgroup in relation to ADOS-CSS score and psychometric scores (* indicates statistically significant results): F = stands for female; M = stands for male. ADOS-CSS: ADOS Calibrated Severity Score; PIQ: Performance Intelligence Quotient; CBCL_INT: Child Behavior Checklist—Internalizing Problems Scale; CBCL_EXT: Child Behavior Checklist—Externalizing Problems Scale; CBCL_TOTAL: Child Behavior Checklist—Total Problems Scale; CBCL_ED: Child Behavior Checklist—Emotional Dysregulation Profile.

	Mean Score		*p*-Value	Cohen’s d
ADOS-CSS	F = 6.35	M = 6.63	0.500	0.159
PIQ	F = 75.33	M = 94.54	0.004 *	0.790
CBCL_INT	F = 60.08	M = 60.43	0.889	0.033
CBCL_EXT	F = 54.69	M = 54.76	0.967	0.009
CBCL_TOTAL	F = 58.58	M = 58.17	0.864	0.038
CBCL_ED	F = 168.69	M = 170.26	0.663	0.096

**Table 11:** Correlation between mean PIQ Score and EEG findings during sleep (* indicates statistically significant results; **^#^** indicates potential trends): 0 = indicates the absence of the corresponding abnormal EEG finding; 1 = indicates the presence of the corresponding abnormal EEG finding. ABNORMAL_EEG_S: abnormal EEG tracing during sleep; PIQ: Performance Intelligence Quotient; EEG_ED_S: epileptiform discharges during sleep; EEG_SLOW_S: slow abnormalities during sleep.

	General		Females		Males	
p(ABNORMAL_EEG_S, PIQ)	0.100 ^#^		0.013 *		0.987	
Cohen’s d(ABNORMAL_EEG_S, PIQ)	0.357		1.127		0.004	
Mean PIQ score as regards ABNORMAL_EEG	0 = 93.46	1 = 85.01	0 = 90.45	1 = 61.33	0 = 94.40	1 = 94.48
p(EEG_ED_S, PIQ)	0.388		0.088 ^#^		0.488	
Cohen’s d(EEG_ED_S, PIQ)	0.191		0.747		0.177	
Mean PIQ score as regards EEG_ED	0 = 91.31	1 = 86.70	0 = 85.25	1 = 64.36	0 = 93.13	1 = 96.53
p(EEG_SLOW_S, PIQ)	0.333		0.491		0.288	
Cohen’s d(EEG_SLOW_S, PIQ)	0.297		0.809		0.361	
Mean PIQ score as regards EEG_SLOW	0 = 90.57	1 = 83.38	0 = 77.48	1 = 52.00	0 = 95.75	1 = 88.61

**Table 12:** Correlation between the clinical scores and the location of epileptiform abnormalities: the PIQ scores in the male subgroup and the entire female subsample were not evaluable due to a lack of participants. Patients showing both focal/multifocal and diffuse epileptiform discharges were excluded from the sample. F-M = stands for frontal midline region; T = stands for temporal region; P = stands for posterior region; D = stands for diffuse discharges. ADOS-CSS: ADOS Calibrated Severity Score; PIQ: Performance Intelligence Quotient; CBCL_INT: Child Behavior Checklist—Internalizing Problems Scale; CBCL_EXT: Child Behavior Checklist—Externalizing Problems Scale; CBCL_TOTAL: Child Behavior Checklist—Total Problems Scale; CBCL_ED: Child Behavior Checklist—Emotional Dysregulation Profile; NE: not evaluable.

	General				Males			
p(ADOS-CSS)	0.549				0.325			
ω^2^(ADOS-CSS)	−0.017				0.004			
Mean ADOS-CSS score	F-M = 6.56	T = 6.22	P = 5.33	D = 6.74	F-M = 6.53	T = 5.87	P = 6.00	D = 7.07
p(PIQ)	0.713				NE			
ω^2^(PIQ)	−0.022				NE			
Mean PIQ score	F-M = 89.94	T = 90.81	P = 109.00	D = 83.00	F-M = 96.70	T = 97.79	P = 126.00	D = 77.80
p(CBCL_INT)	0.777				0.918			
ω^2^(CBCL_INT)	−0.036				−0.063			
Mean CBCL_INT score	F-M = 61.52	T = 58.89	P = 61.67	D = 62.84	F-M = 61.41	T = 59.37	P = 61.00	D = 62.50
p(CBCL_EXT)	0.469				0.754			
ω^2^(CBCL_EXT)	0.007				−0.041			
MeanCBCL_EXT score	F-M = 55.32	T = 52.22	P = 59.33	D = 57.75	F-M = 55.00	T = 53.25	P = 57.50	D = 57.33
p(CBCL_TOTAL)	0.658				0.898			
ω^2^(CBCL_TOTAL)	−0.017				−0.052			
Mean CBCL_TOTAL score	F-M = 59.52	T = 56.00	P = 61.67	D = 61.50	F-M = 59.95	T = 56.62	P = 60.00	D = 60.67
p(CBCL_ED)	0.497				0.818			
ω^2^(CBCL_ED)	−0.007				−0.047			
Mean CBCL_ED score	F-M = 169.56	T = 168.44	P = 172.67	D = 176.55	F-M = 170.05	T = 170.50	P = 175.50	D = 175.27

## Data Availability

The data presented in this study are available upon request from the corresponding author. The data are not publicly available due to privacy or ethical restrictions.

## Supplementary Materials

The following supporting information can be downloaded at: https://www.mdpi.com/article/10.3390/jcm14020529/s1, Supplementary File S1 includes: Table S1. Normality testing of the distribution of the quantitative variables analyzed in our cohort. Table S2: Contingency tables for χ^2^ tests regarding the relationship between slow abnormalities and both sex (see A) and seizures (see B). Table S3: Correlation analysis within the overall cohort and the two sex subcohorts. Figure S1: The distribution of the CBCL-EXT score, CBCL_INT score, and CBCL_ED score within the cohort.

## Abbreviations

ABNORMAL_EEG	Abnormal EEG tracing
ADOS-2	Autism Diagnostic Observation Schedule-2
ADOS-CSS	ADOS Calibrated Severity Score
ADOS-G	Autism Diagnostic Observation Schedule-Generic
ASMs	Anti-seizure medications
APA	American Psychiatric Association
ASD	Autism spectrum disorder
CASP7	Caspase-7
CBCL	Child Behavior Checklist
CBCL 1½-5	Child Behavior Checklist 1½-5
CBCL-ED	Child Behavior Checklist—Emotional Dysregulation Profile
CBCL-EXT	Child Behavior Checklist—Externalizing Problems Scale
CBCL-INT	Child Behavior Checklist—Internalizing Problems Scale
CBCL-TOTAL	Child Behavior Checklist—Total Problems Scale
CHD5	Chromodomain helicase DNA-binding protein 5
CNTNAP2	Contactin-associated protein 2
CYFIP1	Cytoplasmic FMRP-interacting protein 1
D	Diffuse discharges
DR	Drowsiness
DSM-5	Diagnostic and Statistical Manual of Mental Disorders, 5th Edition
DSM-IV-TR	Diagnostic and Statistical Manual of Mental Disorders, 4th Edition, Text Revision
EEG	Electroencephalogram
EEG_ED	Epileptiform discharges
EEG_FAST	Fast abnormalities
EEG_SLOW	Slow abnormalities
EMG	Electromyographic
F	Females
f	Frequency
F-M	Frontal midline brain region
Full-scale IQ	Full-scale intelligence quotient
HMGB1	High mobility group box-1 protein
HV	Hyperventilation
ID	Intellectual disability
ILAE	International League Against Epilepsy
IPS	Intermittent photic stimulation
IQ	Intelligence quotient
M	Males
MN	Mean
N	Number of observations
n	Number of participants
NDD	Neurodevelopmental disorder
NREM	Non-rapid eye movement
*p*	*p* value
P	Posterior brain region
PIQ	Performance intelligence quotient
S	Sleep
SD	Standard deviation
SEAs	Subclinical electroencephalographic abnormalities
SEDs	Subclinical epileptiform discharge
SWA	Slow-wave activity
T	Temporal brain region
W	Wakefulness
WPPSI-III	Wechsler Preschool and Primary Scale of Intelligence—Third Edition
