# Supplementary material for: EEG Abnormalities and Phenotypic Correlates in Preschoolers with Autism Spectrum Disorder: A Single-Center Study
Source: J Clin Med. 2025 Jan 15;14(2):529. doi: 10.3390/jcm14020529 (PMC11766335; doi:10.3390/jcm14020529)
Supplement: Supplementary file 1 [file jcm-14-00529-s001.zip › jcm-3369614-supplementary.pdf]

SUPPLEMENTARY FILE S1

SUPPLEMENTARY TABLES

Table S1. Normality testing of the distribution of the quantitative variables analysed in our cohort: for each parameter we reported the number of observations (N), the mean (MN), the standard deviation (SD) and the p-value (*p*) calculated by the D'Agostino-Pearson normality test (* indicates statistically significant results and non-normal distribution): our sample was large enough that non-normally distributed parameters did not pose a major issue for statistical analysis.
ADOS-CSS: ADOS Calibrated Severity Score; PIQ: Performance Intelligence Quotient; CBCL_INT: Child Behavior Checklist - Internalizing problems scale; CBCL_EXT: Child Behavior Checklist - Externalizing problems scale; CBCL_TOTAL: Child Behavior Checklist - Total problems scale; CBCL_ED: Child Behavior Checklist - Emotional Dysregulation Profile.

PARAMETER DISTRIBUTION		N	MN	SD	*p*
**Age at first EEG**		140	42.75	18.36	<0.001*
**ADOS-CSS score**		135	6.57	1.65	0.413
**PIQ score**		95	89.68	23.18	0.005*
**CBCL_INT score**		133	60.36	9.59	0.403
**CBCL_EXT score**		140	54.75	8.20	0.263
**CBCL_TOTAL score**		140	58.24	10.34	0.314
**CBCL_ED score**		140	169.97	16.35	<0.001*

.

Table S2. Contingency tables for χ^2 tests regarding the relationship between slow abnormalities and both sex (see **A**) and seizures (see **B**). TOT: total; F: female; M: male; EEG_SLOW: slow abnormalities.

A

		Absence of EEG_SLOW	Presence of EEG_SLOW	**TOT**
F		22	4	26
M		94	20	114
**TOT**		116	24	140

B

		Absence of EEG_SLOW	Presence of EEG_SLOW	**TOT**
No seizures		108	23	131
Seizures		8	1	9
**TOT**		116	24	140

Table S3. Correlation analysis within the overall cohort and the two sex subcohorts: the correlation strength is expressed by Pearson's r correlation coefficient. ADOS-CSS: ADOS Calibrated Severity Score; PIQ: Performance Intelligence Quotient; CBCL_INT: Child Behavior Checklist - Internalizing problems scale; CBCL_EXT: Child Behavior Checklist - Externalizing problems scale; CBCL_TOTAL: Child Behavior Checklist - Total problems scale; CBCL_ED: Child Behavior Checklist - Emotional Dysregulation Profile; ABNORMAL_EEG: abnormal EEG tracing; EEG_ED: epileptiform discharges; EEG_SLOW: slow abnormalities.

**TOTAL COHORT**	ADOS_CSS_SCORE	PIQ_SCORE	CBCL_INT_SCORE	CBCL_EXT_SCORE	CBCL_TOTAL_SCORE	CBCL_ED_SCORE	ABNORMAL_EEG	EEG_ED	EEG_SLOW
ADOS_CSS_SCORE	–								
PIQ_SCORE	-0.2164154	–							
CBCL_INT_SCORE	0.1805279	-0.1941960	–						
CBCL_EXT_SCORE	-0.0045722	-0.1667937	0.7606199	–					
CBCL_TOTAL_SCORE	0.0667340	-0.2011858	0.9289404	0.8944841	–				
CBCL_ED_SCORE	0.0134443	-0.1542865	0.7715920	0.8584089	0.8537503	–			
ABNORMAL_EEG	-0.0321286	-0.1893755	0.0749508	0.1036954	0.1128386	0.1213715	–		
EEG_ED	-0.0213339	-0.1267739	0.0733073	0.1097505	0.1056678	0.1214619	0.8790491	–	
EEG_SLOW	-0.0803306	-0.0979562	0.0989674	-0.0069306	0.0735979	0.0610777	0.4114353	0.1646828	–

**FEMALE COHORT**	ADOS_CSS_SCORE	PIQ_SCORE	CBCL_INT_SCORE	CBCL_EXT_SCORE	CBCL_TOTAL_SCORE	CBCL_ED_SCORE	ABNORMAL_EEG	EEG_ED	EEG_SLOW
ADOS_CSS_SCORE	–								
PIQ_SCORE	-0.3378690	–							
CBCL_INT_SCORE	0.2146336	-0.3527403	–						
CBCL_EXT_SCORE	-0.0059222	-0.2020504	0.8582671	–					
CBCL_TOTAL_SCORE	0.1123055	-0.3096573	0.9502734	0.9096948	–				
CBCL_ED_SCORE	-0.0150429	-0.2451476	0.8089441	0.8388270	0.8701070	–			
ABNORMAL_EEG	-0.0785247	-0.3185022	0.2636246	0.3814593	0.3052619	0.2971137	–		
EEG_ED	-0.0478383	-0.1582840	0.1942078	0.2774997	0.1594641	0.1478832	0.8496618	–	
EEG_SLOW	-0.0211307	-0.1444526	-0.0127824	0.0746786	0.0653938	0.0875114	0.3102526	-0.0663906	–

**MALE COHORT**	ADOS_CSS_SCORE	PIQ_SCORE	CBCL_INT_SCORE	CBCL_EXT_SCORE	CBCL_TOTAL_SCORE	CBCL_ED_SCORE	ABNORMAL_EEG	EEG_ED	EEG_SLOW
ADOS_CSS_SCORE	–								
PIQ_SCORE	-0.1526091	–							
CBCL_INT_SCORE	0.1674538	-0.1121247	–						
CBCL_EXT_SCORE	-0.0054979	-0.1640315	0.7436925	–					
CBCL_TOTAL_SCORE	0.0537657	-0.1437806	0.9251833	0.8939551	–				
CBCL_ED_SCORE	0.0173728	-0.1388478	0.7660965	0.8635765	0.8519782	–			
ABNORMAL_EEG	-0.0105610	-0.0754055	0.0279540	0.0508609	0.0671068	0.0892096	–		
EEG_ED	-0.0057275	-0.0537216	0.0422271	0.0768720	0.0915821	0.1202611	0.8842891	–	
EEG_SLOW	-0.1006656	-0.1101261	0.1295473	-0.0227887	0.0760493	0.0545194	0.4375950	0.2174118	–

SUPPLEMENTARY FIGURES

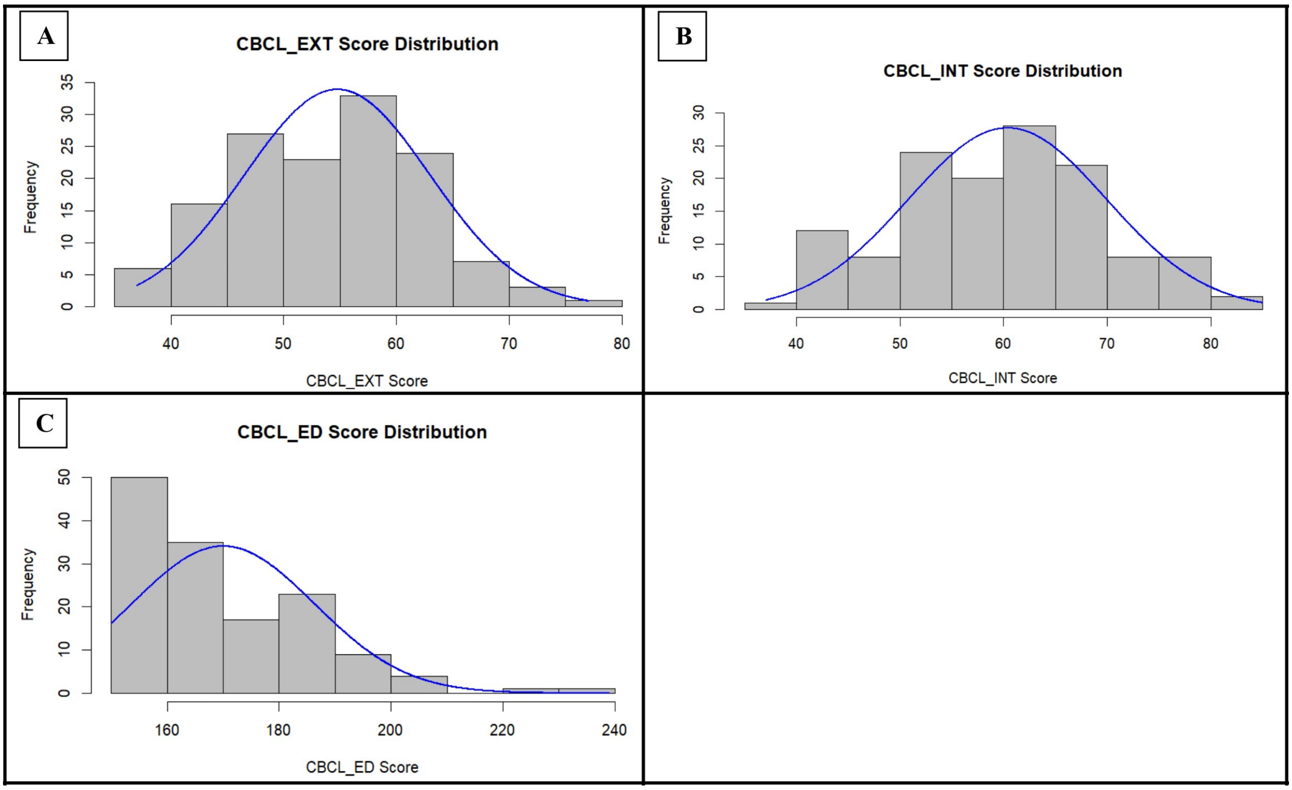

Figure S1. Distribution of CBCL-EXT score, CBCL_INT score and CBCL_ED score within the cohort: for each parameter, the normal curve expected for those values of mean and standard deviation is indicated in blue.

CBCL_EXT: Child Behavior Checklist - Externalizing problems scale; CBCL_INT: Child Behavior Checklist - Internalizing problems scale; CBCL_ED: Child Behavior Checklist - Emotional Dysregulation Profile.
